# Climate-Related Pathways to Child Stunting: Implications for Community Health Nursing - A Scoping Review

**DOI:** 10.12688/f1000research.181964.1

**Published:** 2026-05-21

**Authors:** Akhmadi Akhmadi, Muhamad Abi Zakaria, Ayu Anita, Eiren Hotdi Huriana Siregar, Berliando Toro Betty Runesi, Arif Annurahman, Dhiana Ayu Novitasari

**Affiliations:** 1 Gadjah Mada University Department of Mental Health and Community Nursing, Yogyakarta, Special Region of Yogyakarta, Indonesia; 2 Master of Nursing, Gadjah Mada University, Yogyakarta, Special Region of Yogyakarta, Indonesia

**Keywords:** Stunting, Climate Change, Community Health Nursing, PCC Framework

## Abstract

**Background:**

Climate change is a key factor exacerbating the incidence of stunting in children. Various climate events, such as droughts, floods, extreme temperatures, and disruptions to the growing season, lead to reduced food security, lower agricultural productivity, and diminished access to nutritious food and clean water. In this context, strengthening community-based health services is crucial, given their role in monitoring child growth, providing nutritional counseling, and facilitating referrals—all of which contribute to efforts to reduce stunting.

**Aim:**

This review aims to synthesize scientific evidence regarding the relationship between exposure to climate change and the risk of stunting, as well as the recommended policies. This will subsequently be reflected in community-based nurse services to support the development of more integrated and climate-responsive public health strategies.

**Methods:**

A scoping review was conducted using the PCC framework: Population (children under five years old), Concept (climate-related determinants of stunting), and Context (within a global scope). Searches were conducted across PubMed, ScienceDirect, Scopus, ProQuest, and Web of Science, supplemented by manual searches in January 2026. Of the 5,113 identified articles, 763 articles were screened based on title and abstract, and ten articles met the inclusion criteria after full-text review. Included studies were original research published between 2016 and 2026 that addressed climate change and stunting. Study quality was assessed using the Joanna Briggs Institute checklist, and thematic analysis was applied to synthesize the findings.

**Results:**

Three principal themes were identified: the direct impact of climate change on child growth and stunting; the indirect impact through food security, environmental conditions, socioeconomic determinants, health, and regional variations; and policies and climate change adaptation strategies for child nutrition.

**Conclusion:**

Overall, climate change contributes to child stunting through interconnected pathways, highlighting the essential role of community health nurses.

## Introduction

The prevalence of stunting remains a significant global public health problem, particularly in low- and middle-income countries (LMICs). Globally, the prevalence of stunting among children under five years old has shown a gradual decline since 2000, from approximately 33.1% to 22.0% in 2020. ^1, 2^ However, this decline has not been sufficient to substantially reduce the burden of stunting, where approximately 148 million children under five worldwide still experience stunting. ^2^ The burden of these cases is primarily concentrated in regions of Asia and Africa, which are low- and middle-income countries (LMICs). ^1^ In several countries within these regions, the prevalence of stunting remains above the 20% threshold, which the World Health Organization defines as an indicator of a public health problem. ^3^

Historically, stunting has been predominantly viewed as a nutritional problem, largely caused by inadequate food intake and non-optimal infant and young child feeding practices. ^4, 5^ However, increasing evidence indicates that focusing only on nutrition is not enough to significantly reduce stunting ^6^ without addressing broader structural determinants such as poverty, environmental exposure or sanitation, and health system capacity. ^1, 7^ If complex and cross-sectoral determinants are ignored, efforts to address child growth patterns in low- and middle-income countries (LMICs) may be inadequate.

Among these broader structural determinants, climate change has emerged as an increasingly important driver of malnutrition. Climate-related events such as droughts, floods, extreme temperatures, and disruptions to planting seasons disrupt food security, reduce agricultural productivity, and limit access to nutritious food and clean water. ^8, 9^ These disruptions increase the risk of energy and micronutrient deficiencies and increase exposure to infectious diseases such as diarrhea, thereby accelerating growth impairment. ^10, 11^ These impacts disproportionately affect households that are economically and socially disadvantaged, where limited resources and weak access to health systems exacerbate vulnerability to climate shocks. ^12^

Strengthening community health services is highly important, as evidence indicates that community-based health services play an important role in monitoring child growth, providing nutrition counseling, and making referrals that contribute to reducing stunting. ^13, 14^ On the other hand, primary health care systems also face additional pressures due to climate change, including extreme weather and environmental conditions, which have been associated with increased demand for health services, particularly in remote areas. ^15^ Along with the impacts of climate change, community-oriented primary health care services need to adapt through strengthening community resilience, cross-sectoral collaboration, and providing more targeted support.

Although previous studies have examined nutritional intervention, environmental exposures, or health system responses separately, evidence integrating climate-related exposures, stunting risk, and community health system adaptation remains limited. Most studies still focus on nutrition interventions, without adequately considering environmental and structural determinants that affect child growth. Therefore, a more comprehensive understanding is required of how climate change affects the risk of stunting and how community-based health services respond to these challenges. This study aims to synthesize scientific evidence on the association between climate change exposure and stunting risk, as well as the adaptive response of community-based health services in addressing these challenges.

## Methods

### study design

This study used a scoping review design, following the criteria of the *Preferred Reporting Items for Systematic Reviews and Meta-Analyses extension for Scoping Reviews* (PRISMA-ScR). ^16^ In addition, this study obtained ethical approval from the Medical and Health Research Ethics Committee of the Faculty of Medicine, Public Health, and Nursing, Universitas Gadjah Mada–RSUP Dr. Sardjito, with the number: KE/FK/0420/EC/2026.

### Problem identification

The problem identification in this study used the PCC (Population, Concept, Context) approach. ^17^ The population was children under 5 years old, a group at risk of growth impairment. The concept focused on the determinants of stunting in the context of climate change. The context encompassed the global level, particularly low- and middle-income countries and climate-vulnerable regions. Based on the PCC framework, this study aims to identify and map the determinants linking climate change to stunting among children under five and their implications for community health practice. This study addresses three main questions: (1) what are the main determinants that link climate change with stunting, (2) how climate change affects the nutritional status and growth of children, and (3) what the implications for community nursing practice and public health interventions are.

### Eligibility criteria

The inclusion criteria in this study included: (1) studies that discussed the relationship between climate change and the incidence of stunting among children under five; (2) articles that identified determinants or mediation/moderation pathways that linked climate change with child nutritional status; (3) studies that included aspects of practice or policy in addressing stunting due to climate change; (4) original research articles with quantitative, qualitative, or mixed methods designs; (5) available in full-text access; (6) were published within the period of January 2016 to January 2026; and (7) written in English. Meanwhile, the exclusion criteria included: (1) articles that only discussed stunting without any association with climate change; (2) studies that focused on traditional interventions without relevance to environmental or community contexts; and (3) non-scientific articles such as editorials, commentaries, or narrative reviews that did not present primary data or systematic synthesis.

### Research strategy

The article search was conducted by three researchers (A, AA, MAZ) using predetermined criteria, with reference to the PCC components. The search strategy was carried out in three stages: an initial search in at least two relevant online databases, followed by analysis of keywords and index terms from the initial results to refine the search strategy and make it more comprehensive across all identified databases. The search was conducted in January 2026 through the PubMed, ScienceDirect, Scopus, ProQuest, and Web of Science databases using Boolean operators (AND/OR) to combine relevant keywords. In addition to database searching, a general web search was performed through search engines (e.g., Google Scholar) and other sources of grey literature. This step aimed to identify potentially relevant studies that were not indexed in the selected databases, thereby enhancing the comprehensiveness of the evidence base. The keywords included topics related to stunting and child growth problems, as well as environmental aspects such as climate change, food security, and sanitation. The combination of keywords was adapted to each database’s system. The third stage included screening the reference lists of all selected sources to identify additional relevant studies. ^18^

### Data extraction

Two researchers (EHHS and BTBR) independently conducted data extraction from the included studies. The data collected included study characteristics such as title, authors, year, objectives, location, design, type of climate change, and results. All data were then summarized and analyzed in accordance with the study objectives and presented according to the themes identified in Table 2.

### Data analysis and synthesis

Thematic synthesis was used to analyze data from the studies included in the review process inductively. ^19^ This process consisted of three stages: coding the extracted data, grouping into descriptive themes, and developing analytical themes that extended beyond the findings reported in the original studies.

### Quality appraisal

The quality appraisal of articles in this integrative review used the critical appraisal tools from the Joanna Briggs Institute (JBI) to assess cross-sectional ^20^ and cohort study designs. ^21^ The results of the critical appraisal assessment are presented in Table 1 below.

**Table 1.** JBI critical appraisal.

Author	Checkilst JBI	Yes (%)	No (%)	Unclear (%)	NA
Yeboah et al., 2024 ^27^	Cross-Sectional	100	0	0	0
Mank et al., 2021 ^24^	Cohort	72.73	0	9.09	18.18
Tusting et al., 2020 ^28^	Cross-Sectional	100	0	0	0
Ren et al., 2022 ^31^	Cohort	81.82	0	0	18.18
Dimitrova et al., 2020 ^26^	Cross-Sectional	100	0	0	0
Lloyd et al., 2018 ^30^	Cross-Sectional	50	25	25	0
Ngwira et al., 2020 ^25^	Cross-Sectional	100	0	0	0
Mansfield et al., 2025 ^29^	Cross-Sectional	100	0	0	0
Islam et al., 2025 ^23^	Cross-Sectional	100	0	0	0
Merwe et al., 2022 ^22^	Cohort	73	12	9	0

## Results

### Study selection

**Figure 1.**
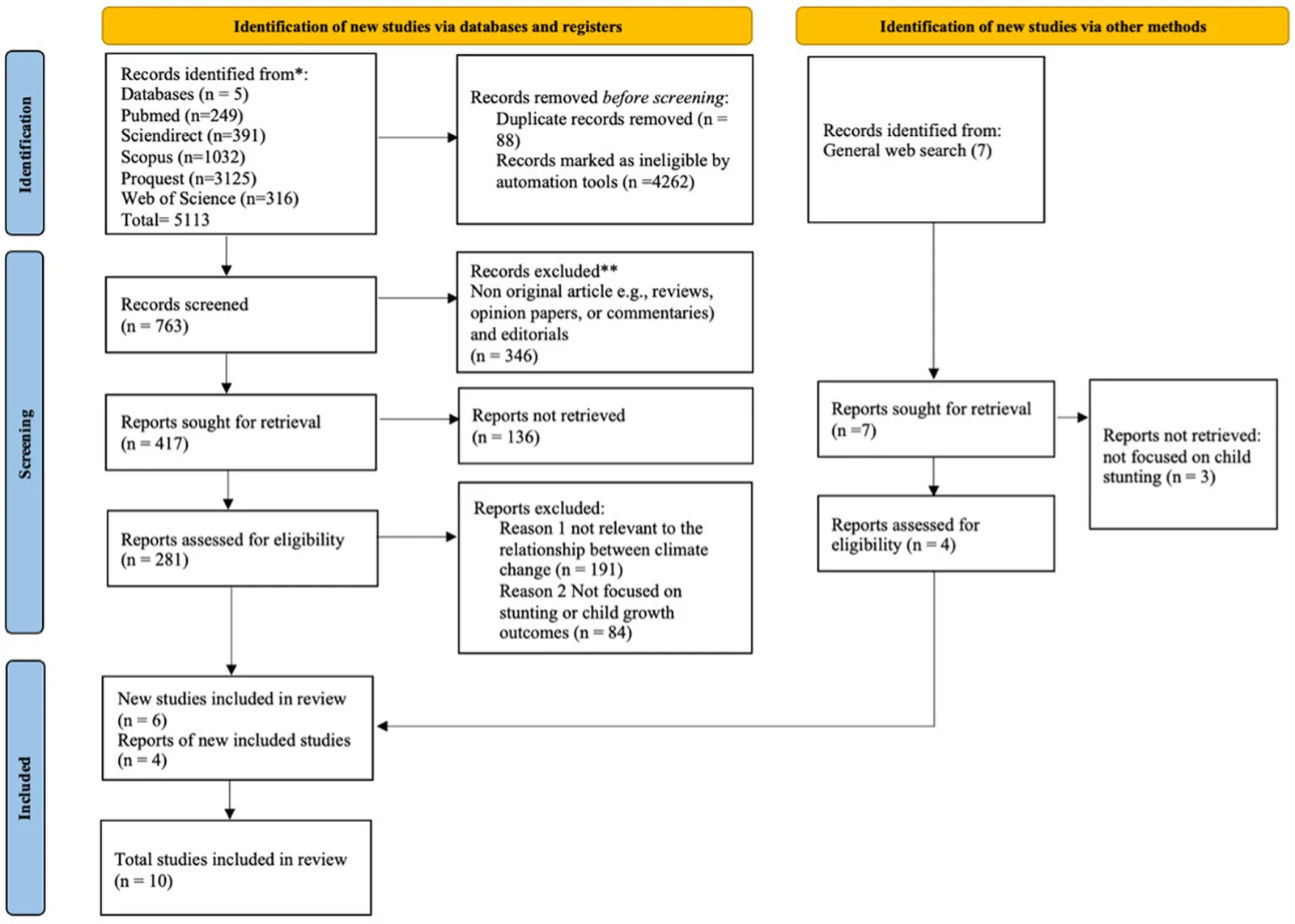
PRISMA flow diagram. ^16^

Two independent researchers (AA and DAN) conducted the identification and screening process using the Rayyan AI tool. In the initial stage, a total of 5,113 articles were identified from five main electronic databases, namely PubMed, ScienceDirect, Scopus, ProQuest, and Web of Science. In addition, manual searching (hand searching) was conducted on Google Scholar and other relevant websites, yielding an additional 7 articles; 3 of these were excluded because they were not focused on child stunting. Subsequently, the initial screening was conducted on 763 articles based on titles and abstracts, with 346 excluded as non-original research, such as review articles, opinions, commentaries, and editorials. A total of 281 articles were then assessed for eligibility through full-text review; however, 275 articles were excluded for the following reasons: not relevant to the relationship between climate change and stunting in children (n = 191) and not directly discussing stunting or child growth disorders (n = 84). Lastly, 10 articles were included in this review, comprising seven from the main screening and three from manual searching. The entire process is presented in Figure 1, namely the PRISMA flow diagram.

### Study characteristics

To understand how climate change affected the incidence of stunting in children, a review of several empirical studies from various countries with varying levels of climate change exposure was conducted. These studies differed in research design, geographic scope, and the types of climate variables examined, including rainfall anomalies, extreme temperatures, and seasonal variations. Most studies used a cross-sectional design (n = 7), while several others used a cohort approach to strengthen causal relationships (n = 3). The main characteristics of each study, including title, authors, year, country, design, type of climate change, and key findings, are presented in Table 2.

**Table 2.** Study characteristics.

First author (Year)	Purpose	Country	Design	Type of climate change	Result
Yeboah et al., 2024 ^27^	To assess the quality of nutritional status assessment in primary care and its role in moderating the impact of rainfall deviation on child growth	Burkina Faso	Cross-sectional	Rainfall deviation	Child growth is hampered by rainfall deviation, but high-quality nutrition services can reduce its negative impact, although the benefits are less noticeable in boys and poor families
Mank et al., 2021 ^24^	To explore how rainfall variability affects children’s dietary patterns and its relationship to nutritional status	Burkina Faso	Cohort	Rainfall variability	Rainfall variability affects dietary patterns and is associated with a deterioration in children’s nutritional status
Tusting et al., 2020 ^28^	To assess the relationship between environmental temperature and stunted growth in young children in various sub-Saharan African countries	Sub-Sahara Africa	Cross-sectional	Soil surface temperature	Extremely high or low environmental temperatures are associated with an increased risk of stunting and malnutrition in children
Ren et al., 2022 ^31^	To evaluate the effects of exposure to extreme temperatures during pregnancy on the risk of preterm birth and its subtypes in several cities in China	China	Cohort	Extreme temperatures (heat and cold)	Exposure to extreme temperatures during pregnancy increases the risk of preterm birth, with variations in effects between cities influenced by local economic conditions and health services
Dimitrova et al., 2020 ^26^	To assess the impact of monsoon anomalies on child stunting and identify different demographic vulnerabilities	India	Cross-sectional	Rainfall anomalies (flooding)	Exposure to extreme rainfall during pregnancy and infancy increases the risk of stunting, with varying vulnerability according to gender, social group, mother’s education level, and hygiene practices
Lloyd et al., 2018 ^30^	To assess how climate change impacts income and food prices, which will affect the prevalence of stunting in children under 5 years of age	Multiple countries	Cross-sectional	Climate	Climate change is projected to increase the number of stunted children, especially in rural areas; the interaction between low income and rising food prices moderates the risk of stunting, with a greater effect in low-income countries with high food prices
Ngwira et al., 2020 ^25^	To investigate the influence of climate variables (rainfall, temperature, length of rainy season) and spatial location on stunting, wasting, and overweight in children under 5 years of age	Malawi	Cross-sectional	Rainfall, temperature, rainy season, water sources, vegetation	Rainfall increases the risk of stunting and overweight, rising temperatures are associated with increased wasting, length of rainy season and vegetation moderate risk; there is a slight positive correlation between stunting and overweight, as well as spatial variation in the risk of wasting and underweight
Mansfield et al., 2025 ^29^	To examine the association between temperature anomalies (heat and cold) and child stunting and assess the role of household infrastructure and socio-demographic factors.	Burkina Faso and Kenya	Cross-sectional	Temperature anomalies (heatwaves and cold anomalies)	Heat anomalies increased stunting risk in Kenya but decreased it in Burkina Faso, while cold anomalies slightly increased stunting in both countries. Electricity access was protective in both countries, while sanitation, water access, and household wealth reduced stunting mainly in Kenya
Islam et al., 2025 ^23^	To analyze the relationship between children’s nutritional status and social, economic, environmental, and climatic factors	Pakistan	Cross-sectional	Temperature, precipitation, soil moisture	Socioeconomic factors, food security, and maternal status are positively associated with stunting. Rising temperatures and increased rainfall are Anegatively associated with stunting and increase the risk of wasting; they are also linked to diarrhea and high parity
Merwe et al., 2022 ^22^	To analyze the impact of climate change (temperature and rainfall) on child malnutrition, particularly stunting and underweight.	Nigeria	Cohort	Temperature and precipitation	Rising temperatures are significantly associated with increased rates of stunting and underweight. Decreased rainfall also increases the risk of malnutrition (indirectly). The impact is greater in rural areas than in urban areas

### Synthesis

This review analyzed evidence from seven studies that examined how climate change affected child malnutrition, particularly stunting, both directly through environmental exposures (such as temperature and rainfall anomalies) and indirectly through disruptions to economic systems and food security. These studies covered various geographic regions, including Sub-Saharan Africa, South Asia, East Asia, as well as global-level analyses. The following synthesis table ( Table 3) presents the main subthemes and codes from the reviewed studies to illustrate the core findings within each thematic category.

**Table 3.** Synthesis.

Code	Subthemes
**The direct impact of climate change on child growth and stunting**	
Rainfall variability increases stunting risk ^24– 26^	Temperature, rainfall, and correlations with anthropometric measures.
A 1 °C increase in temperature and a 1 mm increase in rainfall are associated with a decline in LAZ scores ^23^	
A 1 °C rise in temperature increases the risk of stunting in both urban and rural areas ^22^	
Rainfall deviations increase stunting risk, though this can be mitigated by the quality of healthcare services ^27^	
Daytime temperatures above 35 °C are associated with reduced odds of stunting among children aged 2–5 years ^28^	
Hot and cold temperatures cause physiological stress ^29^	Physiological mechanisms resulting from climate change.
High temperatures increase the risk of dehydration ^23^	
**Indirect impacts of climate change on stunting**	
Temperature may disrupt food availability, cause drought, reduce soil quality, and decrease water availability ^29^	Disruptions in food security, environment, and sanitation.
Rising extreme temperatures may reduce agricultural yields due to dry soil conditions, thereby potentially disrupting food availability and children’s nutritional intake ^23^	
Declining rainfall may worsen agricultural outcomes and water availability ^22^	
Rainfall and season length improve food production, while temperature affects aridity, which in turn influences food security ^25^	
Rainfall variability affects food availability and consumption patterns, particularly during lean seasons ^24^	
Climate change contributes to declining incomes among the poorest groups and alters food prices relative to household income ^30^	Household economic changes.
The impacts of climate change on stunting are more severe among socially vulnerable groups ^26^	
Rainfall influences families’ ability to purchase food ^24^	
Extreme temperatures may increase the incidence of diseases such as diarrhea and malaria ^23, 29^	Increased risk of infectious diseases and maternal child health disruptions.
Heat exposure during pregnancy may impair fetal growth ^23^	
Changes in rainfall patterns, including droughts and floods, may disrupt access to clean water and sanitation, thereby increasing the risk of infectious diseases ^23^	
Extreme rainfall is associated with a higher risk of infectious diseases ^23^	
Extreme heat may cause dehydration, increased blood viscosity, and reduced uterine blood flow, while extreme cold may increase blood viscosity, vasoconstriction, and infection risk—all of which may trigger preterm birth ^31^	
Children in rural areas are more vulnerable to climate change–related stunting because they are more dependent on agriculture ^22^	Regional differences in climate change impacts.
The relationship between temperature anomalies and stunting varies across countries ^29^	
The effects of extreme temperatures differ across cities ^31^	
**Policies and climate change adaptation strategies for child nutrition**	
Climate-friendly policies are needed through improved infrastructure, electricity access, education, and adaptive agriculture ^22^	Multisectoral and locally contextualized adaptation policies.
Policies should be tailored to local contexts ^29^	
Strengthening primary healthcare systems through early detection and management of nutritional problems ^27^	
Nutritional interventions should prioritize pregnant women and infants, alongside strengthening women’s education ^26^	Interventions for vulnerable groups (mothers and children).
Protection measures for pregnant women and infants ^31^	
Public health strategies such as providing cooler housing environments ^28^	Environment-based health interventions.
Adaptation through food diversification and home gardening ^24^	
Stunting interventions should consider climatic factors such as rainfall, temperature, and seasonality, and should be implemented equitably across all regions ^25^	

### Theme 1. The direct impact of climate change on child growth and stunting

**Temperature, rainfall, and anthropometry correlation**, variations in climate determinants, particularly temperature and rainfall, have been shown to affect the incidence of stunting. A 1 °C increase in temperature was associated with a decrease in children’s LAZ scores. ^22, 23^ Variations in rainfall were also reported to increase the prevalence of stunting, ^24– 26^ more specifically, ^23^ reported that a 1 mm increase in rainfall was associated with a 0.1 percentage point increase in stunting. These impacts could be reduced by the quality of health services. ^27^ However, findings indicate that daytime temperatures above 35 °C are associated with reduced odds of stunting among children aged 2–5 years, ^28^ likely due to differences in adaptive physiological responses at this age group.

**Physiological mechanisms due to climate change,** Exposure to both extreme heat and cold has been shown to cause physiological stress that disrupts child growth. ^29^ Children have lower heat tolerance than adults; therefore, high temperatures increase the risk of dehydration, leading to a significant decrease in HAZ scores. ^23^

### Theme 2. The indirect impact of climate change on stunting

**Disruptions in food security, environment, and sanitation**: Temperature and rainfall anomalies due to climate change are known to disrupt food security through reduced agricultural yields, degradation of soil quality, as well as decreased water availability, which affect child nutritional intake. ^22, 23, 29^ In addition, rainfall and season length affect food production levels through changes in aridity, which have implications for household food security. ^25^ Variations in rainfall also affect food availability and community consumption patterns, particularly during lean seasons. ^24^

**Household economic changes**: Climate change can lead to declines in the income of the poorest groups ^26^ and contributes to increases in food prices relative to household income, thereby reducing families’ ability to access nutritious food. ^30^ In addition, the impact of rainfall on stunting is affected by socioeconomic determinants, including households’ ability to obtain food from the market, which is highly dependent on financial conditions. ^24^

**Increased risk of infectious diseases and maternal health disorders:** High temperatures are associated with an increased incidence of diseases such as diarrhea and malaria, as well as dehydration, which affect child growth and may even affect fetal growth during pregnancy. ^23, 29^ In addition, changes in rainfall, such as droughts and floods, can disrupt access to clean water and sanitation, thereby increasing the risk of infectious diseases and hindering child growth. ^23, 26^ Physiologically, exposure to extreme heat and cold can disrupt body systems, including changes in blood flow, increased blood viscosity, and an increased risk of infection, leading to preterm birth and impaired child growth. ^31^

**Regional variations in the impact of climate change,** Children in rural areas are more vulnerable to stunting due to a higher dependence on the agricultural sector, which is sensitive to climate change. ^22^ In addition, the impact of temperature changes on stunting also shows inconsistent patterns across countries, both for high and low temperatures. ^29^ In a more specific context, the impact of extreme temperatures also differs across cities, indicating that regional contextual determinants determine the magnitude of climate-change-related stunting risk. ^31^

### Theme 3. Policies and climate change adaptation strategies for child nutrition

**Multisectoral and locally contextualized adaptation policies**: Climate-friendly policies are required to reduce the long-term impacts of climate change on child malnutrition, including through improvements in infrastructure, expansion of electricity access, strengthening of the education sector, and the development of adaptive agriculture. ^22^ In addition, stunting intervention policies need to be adjusted to local contexts, given that differences in socioeconomic and environmental conditions across regions can affect the effectiveness of interventions. ^29^ On the other hand, strengthening primary health care systems also becomes an important component in responding to the impacts of climate change on child nutritional status, particularly through early detection and quick and sustained management of nutritional problems. ^27^

**Interventions for vulnerable groups (mothers and children)**: Nutrition interventions should be prioritized for vulnerable groups, particularly pregnant women and infants, and accompanied by strengthening women’s education to reduce vulnerability to the impacts of climate change. ^26^ In addition, special protection for pregnant women and infants is important for addressing the impacts of climate change, as this group is at higher risk of health disorders and impaired nutritional status due to environmental pressures. ^31^

**Environment-based health interventions,** Public health strategies need to consider the impact of high temperatures on child growth, for example, through the provision of cooler housing. ^28^ Climate change adaptation is also important to maintain food security and health, such as through food diversification and home gardening. ^24^ In addition, stunting interventions need to consider climate determinants such as rainfall, temperature, and seasons, and be implemented equitably across regions. ^25^

## Discussion

This review synthesizes scientific evidence on the relationship between climate change exposure and the risk of stunting, as well as the role of community-based health services in supporting the development of integrated, climate-responsive public health strategies. Overall, the literature shows that climate change, particularly temperature and rainfall, is associated with stunting through various pathways directly through physiological changes and anthropometric growth indicators, and indirectly through changes in environmental and sanitation conditions, disruptions in food security, and an increased risk of infectious diseases. The findings also show variations in impacts based on geographic regions and vulnerable population groups. In addition, several studies highlight the importance of policy analysis in responding to the impacts of climate change on child nutritional status.

Evidence suggests that various studies show that climate change can directly affect child growth, as evidenced by anthropometric measurements of height-for-age. ^22– 26^ This is caused by physiological stress and an increased risk of dehydration under climate change. ^23, 29^ Dehydration in children may result from elevated ambient temperatures that increase fluid loss. Children’s thermoregulatory capacity and body fluid reserves are still immature, making them more vulnerable to fluid imbalance that can disrupt metabolism and growth. ^32, 33^ However, one identified study reported that increased temperature is associated with a decrease in the incidence of stunting. This finding may be explained by physiological responses to heat that develop along with growth and the maturation of body systems. ^32^

Various studies have also found an association between climate change and stunting through multiple pathways. Climate change causes environmental changes such as reduced soil quality and decreased water availability (aridity), which lead to reduced crop yields, ^22, 23, 25, 29^ particularly during lean periods. ^24^ These climate changes also worsen the quantity and quality of food production, including nutrient and antinutrient composition, as well as nutrient bioavailability. These conditions ultimately reduce the availability of macro- and micronutrients in the global food supply. ^34^ They also contribute to food price instability, thereby affecting households’ ability to provide diverse and nutritious diets. ^24, 26, 30, 35^

Another indirect pathway is that climate change increases the risk of infections, as poor sanitation can increase exposure to fecal pathogens. ^23, 26, 29, 31, 36^ This can trigger chronic intestinal inflammation and reduces the capacity for nutrient absorption, thereby impairing children’s linear growth. ^36, 37^ Recurrent infections also disrupt the immune system and divert the body’s energy from growth processes to disease response. ^36, 38^ In addition, extreme temperatures during pregnancy can disrupt physiological conditions such as dehydration, increased blood viscosity, and impaired blood flow, which can ultimately trigger preterm birth. ^31, 39, 40^ Children who are born prematurely have a twofold higher risk of experiencing stunting, ^41^ because it is associated with low birth weight, ^42^ limited nutrient reserves, ^43, 44^ as well as increased susceptibility to infections. ^45, 46^ Taken together, these determinants can impair children’s linear growth in early life.

This study shows that the impact of climate change on stunting is heterogeneous across regions. Rural communities, which largely depend on rain-fed agriculture and climate-sensitive resources, tend to be more vulnerable to these changes. ^22, 47^ Limited access to health services in rural areas can also influence the relationship between climate determinants and stunting. ^48^ This results in inconsistent patterns between temperature anomalies and stunting incidence. ^29, 31^

The analysed studies also emphasize the importance of integrated and climate-adaptive policies is also emphasized. ^22, 29^ This highlights a broader scientific responsibility, as the studies not only identify the relationship between climate change and stunting but also offer policy solutions to reduce this risk. The recommended policies include strengthening interventions tailored to local needs, such as improving agricultural infrastructure ^49, 50^ and developing adaptive practices such as home gardening. ^41^ In addition, strengthening primary health care services through education and early detection of nutritional problems is key, particularly by prioritizing interventions for pregnant women ^51, 52^ and infants, the most vulnerable groups. ^53^

### Future implications for community health nursing practice

The studies above reflect that climate change has a very close relationship with stunting, operating through both direct and indirect pathways. There are also policy recommendations that can serve as a reference for formulating mitigation strategies. As one of the health problems that affects the long-term quality of life, addressing stunting is essential. Community health nursing needs to contribute to these efforts through the following steps.

### Transformation of community health nurses into “climate-literate” practitioners

Community health nursing practice must adopt cross-sectoral collaboration to integrate early warning systems for nutrition into primary health care. ^54^ By understanding local climate patterns, Community Health Nurses can take anticipatory actions such as providing prophylactic supplementation or strengthening sanitation before periods of extreme climate, to prevent nutritional crises. This step positions Community Health Nurses as frontline actors in protecting human capital from the threats of the global climate crisis. ^55– 57^

### Community-based educational interventions as an implementation strategy

Health education is a key strategy in addressing the impacts of climate change on child stunting at the community level. Community nurses have an important role in increasing family awareness of the risk of stunting caused by climate change, both directly and indirectly. ^58^ Health education that can be provided includes balanced nutrition for pregnant women and children, ^59^ the promotion of good hygiene and sanitation practices, ^60^ and supporting early detection of growth problems through routine monitoring. ^52^ In addition, improving health literacy and maternal education has been shown to reduce vulnerability to malnutrition related to climate change. ^61, 62^

### Health adaptation at family and community level

In addition to education, adaptation strategies are required to strengthen household resilience to climate change. ^63^ Adaptation can be implemented through family-based practices, such as utilizing food resources, ^64^ promoting home gardening, ^65^ and adjusting daily behaviors according to seasonal and environmental conditions. ^24^ These approaches have been shown to improve household food security and dietary diversity, which in turn affect child nutrition. ^66^ Community nurses play a vital role in guiding families to anticipate climate variability, such as changes in temperature and rainfall, through adaptive behaviors and community engagement. ^67^ Globally, community-based adaptation strategies are recommended to protect vulnerable populations, particularly pregnant women and children, from the health impacts of climate change. ^68, 69^ Therefore, health adaptation functions not only as a response mechanism but also as a preventive approach to reduce the risk of stunting in the context of climate change.

This study shows that climate change has a significant and complex relationship with the incidence of stunting among children under five through direct and indirect pathways. The direct pathways involve physiological stress and dehydration due to extreme temperatures and rainfall anomalies. Meanwhile, the indirect pathways are mediated by disruptions to food security, declines in household economic status, and increased risk of infectious diseases such as diarrhea and malaria. These findings emphasize that addressing stunting is no longer sufficient through conventional nutrition interventions alone, but must integrate climate change adaptation strategies. Community health nurses play a crucial role as frontline actors within primary health care systems, conducting early detection of malnutrition in climate-vulnerable areas, providing environmental health education to families, and advocating for climate-friendly, multisectoral policies to protect vulnerable groups (such as pregnant women and infants) from the long-term impacts of the environmental crisis.

### Limitation

This study also has several limitations. Although the initial search yielded a substantial number of articles, only a small proportion of studies specifically examined the relationship between climate change and stunting, as well as its policy implications, indicating that this topic remains an emerging field that requires further investigation. In addition, restricting the review to English-language articles may have resulted in the exclusion of relevant studies published in other languages, particularly from developing countries that are significantly affected by climate change. The variation in study designs, which were predominantly characterized by cross-sectional approaches, also represents a limitation, as such designs are less capable of establishing strong long-term causal relationships compared to longitudinal or cohort studies. Finally, differences in geographical contexts across studies may affect the generalizability of the findings; therefore, their application to other regions should carefully consider local environmental conditions and healthcare system capacities.

### Strength

This study has several key strengths. First, it employed a rigorous methodological approach by adopting the PRISMA-ScR framework alongside the PCC (Population, Concept, Context) strategy, ensuring that the process of literature identification and selection was conducted systematically and transparently. Second, this research offers novelty by specifically linking the issue of climate change to child nutritional status and its implications for community nursing practice an area that has thus far been relatively underexplored in an integrated manner. Third, the literature search was conducted comprehensively across five major international databases PubMed, ScienceDirect, Scopus, ProQuest, and Web of Science covering publications from 2016 to 2026, thereby ensuring that the evidence generated remains relevant to current conditions. In addition, the methodological quality of each included study was assessed using instruments developed by the Joanna Briggs Institute (JBI), which further strengthens the validity and credibility of the synthesized findings.

## Conclusions

This study demonstrates that climate change has a significant and complex association with stunting among children under five years of age through both direct and indirect pathways. The direct pathway involves physiological stress and dehydration resulting from extreme temperatures and rainfall anomalies. Meanwhile, the indirect pathway is mediated by disruptions in food security, declining household economic status, and an increased risk of infectiomultisectoraluch as diarrhea and malaria. These findings underscore that addressing stunting can no longer rely solely on conventional nutritional interventions but must also integrate climate change adaptation strategies. Community health nurses play a crucial role as frontline actors within the primary healthcare system in conducting early detection of malnutrition in climate-vulnerable areas, providing environmental health education to families, and advocating for climate-responsive multisectoral policies to protect vulnerable groups particularly pregnant women and infants from the long-term impacts of the environmental crisis.

^*^Consider, if feasible, reporting the number of records identified from each database or register searched (rather than the total number across all databases/registers).

^**^If automation tools were used, indicate how many records were excluded by humans and how many were excluded by automation tools.

## Data Availability

No data are associated with this article.
