# Supplementary material for: Climate-Related Pathways to Child Stunting: Implications for Community Health Nursing - A Scoping Review
Source: F1000Res. 2026 May 21;15:773. [Version 1] doi: 10.12688/f1000research.181964.1 (PMC13560600; doi:10.12688/f1000research.181964.1)
Supplement: Peer review history

# Reviewer report: Reviewer response for version 1

**DOI:** 10.5256/f1000research.200858.r503079

**Published:** 2026-08-18

**Authors:** Martha Irene Kartasurya

**Affiliations:** 1 Universitas Diponegoro, Semarang, Indonesia

This article talk about an important issue regarding the risk of stunting and climate change. However, some improvement can be done to be a better article.

 In the background, it is better if the authors wrote about the previous reviews, not individual study. Has any review been done on this issue? If it has been done, then explain, why this scoping review is needed. 

 In the methods, sub title eligibility criteria, it is better to delete the exclusion criteria, as the inclusion criteria has already covered the same things in the exclusion criteria. 

 In research strategy, the author mentioned that general web search was performed through search engine (e.g., Google Scholar) and other sources of grey literature. Please mention clearer, so that this literature search is replicable. 

 In quality appraisal, there is one study that resulted in 25% no and 25% unclear. Please add the explanation about the results of the critical analysis in determining the studies which are analyzed, including the reason to include that article in the analysis. 

 The study by Ren et al. is not related to stunting, but about the effects of extreme temperature on the risk of preterm birth in China: A population-based multicenter

 cohort study. Please explain why the authors included this study in the analysis or delete this study in the analysis.

Is the review written in accessible language?

Yes

Are all factual statements correct and adequately supported by citations?

Partly

Are the conclusions drawn appropriate in the context of the current research literature?

Yes

Is the topic of the review discussed comprehensively in the context of the current literature?

Yes

Reviewer Expertise:

Child nutrition, maternal and child health, stunting.

I confirm that I have read this submission and believe that I have an appropriate level of expertise to confirm that it is of an acceptable scientific standard.

# Reviewer report: Reviewer response for version 1

**DOI:** 10.5256/f1000research.200858.r487976

**Published:** 2026-06-17

**Authors:** Chiza Kumwenda

**Affiliations:** 1 University of Zambia, Lusaka, Zambia

The articles is addressing one of the most pertinent issues affecting programming of interventions and policies on child growth, especially in LMICs. The authors have clearly presented the justification for undertaking the study. It's unfortunate that the global community has not currently showed sufficient interest in the area of climate change and child growth, especially stunting. This has been reflected in the low numbers of good quality studies as demonstrated by the authors. Overall, the methodology is also well articulated.

 However, the authors should consider the following points to strengthen the paper:

 1. The authors should synchronize the conclusion on the role of community health nurses and the results, currently it appears the results section do not clearly demonstrate directly or indirectly the role of frontline workers. On the other hand the discussion seems to have more details that are not in the results. Partly, because the results are structured under three themes which may not cover most of what is reflected in the discussion. The authors should shift some materials in the discussion and reflect them in the results to enhance the flow.

 2. The authors should also consider the wording of the discussion section, in some cases the narrative of the discussion appears as recommendations. For instance, under the heading "Transformation of Community Health Nurses into "climate literate " practitioners, the first statement fits into the recommendation section. The other sections may be reviewed accordingly.

 3. The word "infectiomultisectoraluch" seems to be a typological error, if not it would be better to use "classical" altenative. Addtionally, the last sentence of the conclusion is "too" long, the one starting with Community health nurses play a crucial role ................and ending with environmental crisis. The authors should consider rephrasing it.

Is the review written in accessible language?

Yes

Are all factual statements correct and adequately supported by citations?

Yes

Are the conclusions drawn appropriate in the context of the current research literature?

Yes

Is the topic of the review discussed comprehensively in the context of the current literature?

Yes

Reviewer Expertise:

Infant an young child nutrition. Infant and young child growth and develpoment. Sustainable Food systems. Body composition assessment.

I confirm that I have read this submission and believe that I have an appropriate level of expertise to confirm that it is of an acceptable scientific standard.
